# Comparing perspectives: patients’ and health care professionals’ views on spiritual concerns and needs in chronic pain care - a qualitative study

**DOI:** 10.1186/s12913-021-06508-y

**Published:** 2021-05-26

**Authors:** Joël Perrin, Nina Streeck, Rahel Naef, Michael Rufer, Simon Peng-Keller, Horst Rettke

**Affiliations:** 1 University Hospital Zurich, Zurich, Switzerland; 2 University of Zurich, Zurich, Switzerland; 3 Institute for Implementation Science in Health Care, Faculty of Medicine, University of Zurich, Zurich, Switzerland; 4 Centre for Clinical Nursing Science, University Hospital Zurich, Zurich, Switzerland; 5 Department of Psychiatry, Psychotherapy and Psychosomatics, Psychiatric University Hospital Zurich, University of Zurich, Zurich, Switzerland; 6 Faculty of Theology, University of Zurich, Zurich, Switzerland

**Keywords:** Chronic pain, Spirituality, Health personnel, Patients, Qualitative research

## Abstract

**Background:**

The spiritual aspect of care is an often neglected resource in pain therapies. The aim of this study is to identify commonalities and differences in chronic pain patients’ (CPP) and health care professionals’ (HCP) perceptions on the integration of spiritual care into multimodal pain therapy.

**Methods:**

We conducted a qualitative exploratory study with 42 CPPs and 34 HCPs who were interviewed in 12 separate groups in five study centres specialising in chronic pain within German-speaking Switzerland. The interviews were transcribed and subjected to a qualitative content analysis. Findings were generated by juxtaposing and analysing the statements of (a) HCP about HCP, (b) HCP about CPP, (c) CPP about HCP, and (d) CPP about CPP.

**Results:**

Views on spiritual concerns and needs in chronic pain care can be described in three distinct dimensions: function (evaluating the need / request to discuss spiritual issues), structure (evaluating when / how to discuss spiritual issues) and context (evaluating why / under which circumstances to discuss spiritual issues). CPPs stress the importance of HCPs recognizing their overall human integrity, including the spiritual dimension, and would like to grant spiritual concerns greater significance in their therapy. HCPs express difficulties in addressing and discussing spiritual concerns and needs with chronic pain patients. Both parties want clarification of the context in which the spiritual dimension could be integrated into treatment. They see a need for greater awareness and training of HCPs in how the spiritual dimension in therapeutic interactions might be addressed.

**Conclusions:**

Although there are similarities in the perspectives of HCPs and CPPs regarding spiritual concerns and needs in chronic pain care, there are relevant differences between the two groups. This might contribute to the neglect of the spiritual dimension in the treatment of chronic pain.

**Trial registration:**

This study was part of a larger research project, registered in a primary (clinicaltrial.gov: NCT03679871) and local (kofam.ch: SNCTP000003086) clinical trial registry.

## Background

Of the 20% of the European population who experience chronic pain, two thirds rate their chronic pain management as insufficient [1–3]. Many of these patients do not feel understood by their physicians [4], which may negatively affect health outcomes, patient satisfaction and costs [5]. Chronic pain reaches beyond the symptoms experienced, fundamentally affecting the person’s life [6, 7]. The World Health Organization (WHO) recommends a health care model that explicitly acknowledges the spiritual aspects of care in addition to prevalent physical, psychological, and social dimensions [8].

Despite this long-standing counsel, spiritual concerns appear to be a rarely accessed element in current multi-modal pain therapies [9]. This may be due in part to HCPs’ discomfiture with this potential resource [10], and CPPs’ lack of awareness that spiritual matters can be an integral part of health care. However, effective chronic pain therapy requires an acknowledgement of individual preferences as well as active commitment by HCPs and CPPs working together at eye level. This entails integrating the spiritual dimension into pain therapy. While spirituality will hardly offer a solution to all problems, it has the strong potential to address some of them [11–14], including those most significant to the chronic pain patient, such as a constructive interpretation of their illness experience and their overall well-being [15].

As part of a larger interprofessional multi-stage research project in German-speaking Switzerland (2017–2021) we investigated CPPs’ and HCPs’ views on spiritual needs and concerns [16, 17]. In the present study, we juxtaposed and compared CPPs’ and HCPs’ views in relation to the therapeutic process. The aim of the present paper is to identify factors that may constrain or enable CPPs and HCPs to successfully consider, evaluate and include the spiritual dimension in chronic pain therapy.

## Methods

We conducted a qualitative-exploratory study involving CPPs and HCPs who were interviewed in separate groups. Findings were generated by juxtaposing and analysing the statements of a) HCPs about HCPs, b) HCPs about CPPs, c) CPPs about HCPs, d) CPPs about CPPs (Table 3).

### Setting and participants

The data set analysed in this paper consists of audio transcripts from a total of 12 semi-structured interviews: seven with CPPs and five with HCPs, conducted at five study centres in German-speaking Switzerland between October 2017 and May 2018 (Table 1).

**Table 1:** Overview of interviews and study centres

Code	Study centre	Group	Duration	n
**1.1**	**rehabilitation facility** with psychosomatic specialisation	CPP	01:12:16	11
**1.2**		HCP	01:16:51	11
**2.1**	**specialist clinic** with Christian religious background	CPP	01:22:40	9
**2.2**		HCP	01:20:28	10
**3.1**	**specialised practice** for rheumatology and pain treatment	CPP	01:17:38	7
**3.2**		HCP	00:26:52	1
**4.1.1**	**pain ambulatory clinic** at a tertiary acute care hospital	CPP	01:16:14	3
**4.1**		CPP	01:10:08	4
**4.2**		HCP	01:15:03	5
**5.1.1**	**specialised clinic** for acute treatment and rehabilitation	CPP	00:43:05	2
**5.1**		CPP	01:07:40	6
**5.2**		HCP	01:17:34	7

Interviews with CPPs were conducted in five focus groups with 4–11 CPPs (*n* = 42). In one study centre only 2 participants showed up and in another, only 3. They were also interviewed to include their views. However, a second recruitment eventually produced a complete focus group in both study centres. An identical interview procedure was applied to all CPP interviews. The 42 participants in total were between 22 and 80 years old ($\overline{x}$ =51.2). Of these, 28 were women. A pain intensity of ≥5 on a 0–10 point numeric rating scale during the last pain episode was a prerequisite for participation (Table 2). At the time of the interview, participants were asked again about the intensity of their pain in the preceding 2 weeks and indicated a pain intensity between 3 and 10 ($\overline{x}$ =6.587). Religious affiliation was varied: Roman Catholic (*n* = 10), Protestant (*n* = 15), other Christian faith communities (*n* = 7) or Muslim (*n* = 3). Seven stated that they were nondenominational.

**Table 2:** Inclusion / exclusion criteria for CPPs and HCPs

Criteria	CPP	HCP
**Inclusion**	• 18 years of age or older • Sufficient knowledge of German to contribute to the focus group interviews • Confirmed medical and / or nursing diagnosis “Chronic pain” (pain for > = 6 months with an intensity of the last pain episode of > = 5 on the 11-point Numeric Rating Scale [NRS; 0 = no pain, 10 = worst imaginable pain])*.	• 18 years of age or older • Trained physician, clinical psychologist, physiotherapist, occupational therapist or qualified nurse, working in the participating health centre • Work experience with CPP > 1 year
**Exclusion**	• Diagnosis of a life-threatening disease (e.g. cancer), which would likely lead to the introduction of topics concerning the end of life. • Cognitive impairment that would lead to diminished participation in the focus group interviews.	None

For the HCPs, the four focus groups consisted of 5 to 11 professionals (*n* = 34). In addition, one individual interview was conducted, as one study centre was a specialised practice allowing for an individual interview only (*n* = 1). The 34 participants were aged between 24 and 61 ($\overline{x}$ =46.71) of which 22 were women. Most were physicians (*n* = 13), followed by psychologists (*n* = 7), nurses (*n* = 6), occupational therapists (*n* = 4), physiotherapists (*n* = 3), and breath-body therapists (n = 1). Participants’ professional experience with chronic pain patients ranged between 6 months and 20 years ($\overline{x}$ =8.75). It was detected after interview completion only that one single particpant had less than the requested 1 year of work experience with CPP. Since this did not seem to affect the group dynamics in any way we still included said interview in our analysis. Religious affiliations varied: Roman Catholic (*n* = 8), Protestant Reformed (n = 8), and other Christian religious communities (*n* = 10). One participant related being interdenominational, seven expressed being nondenominational.

### Data collection process

Recruitment of HCPs and CPPs was carried out by each study centre’s medical director or by an appointed person (e.g. an assistant or a study nurse). Criteria for study participation are given in Table 2. Potential participants were invited to participate in a pre-scheduled interview. HCPs from the respective professional groups were informed and, if interested, provided with the study information. CPPs were subsequently approached. If interested, the study information was provided. After a final decision, written consent was obtained from all participants. The interview guidelines were handed out for individual preparation along with an anonymous socio-demographic questionnaire to be collected at the interview.

### Data collection

For the data collection, focus group interviews were chosen. They have the advantage over individual interviews in that they allow participants to reflect on other group members’ contributions [18–20].

At the outset of every interview, a definition of *spirituality* was shared with participants: “a person’s relationship to what sustains, inspires, and gives meaning in their lives, and the beliefs, attitudes, and practices associated with it. These can be religious or non-religious“. This rather extensive definition served two purposes: First, an intentionally broad definition was used in order to not exclude persons with a secular worldview [21]. Secondly, it acknowledges the different cultural, spiritual, religious and/or ideological backgrounds that converge in interactions between CPPs and HCPs [22]. Our definition was based on the definition from the European Association for Palliative Care but slightly adapted in order to be more approachable for a diverse target group [23].

In several rounds of discussion, the group of authors created the interview guidelines. They consisted of four questions, which were linguistically adapted to the respective group (HCP/CPP):

- To what extent do you consider existential and spiritual concerns and questions significant in the treatment of chronic pain?
- In your opinion, when and how should patients be approached on this topic? From your point of view, what do experts have to consider?
- (In your opinion,) which issues should be addressed here and which should not?
- What could patients (or you as a patient) contribute to a meaningful conversation on this topic?

In the interviews, these questions formed a starting point for a dynamic interaction between participants. All interviews were jointly moderated by a nurse scientist and a theologian with an expertise in biomedical ethics to allow for a balanced, comprehensive exploration of the research topic in the course of the discussion.

The interviews were digitally recorded and subsequently transcribed verbatim. In the process, they were translated from Swiss German dialect to standard German, omitting all identifying details such as personal names and locations to ensure participant anonymity.

### Data analysis

Using HyperResearch®, a computer-aided qualitative data analysis software for keyword coding the transcripts were analysed according to Mayring’s qualitative content analysis. The aim of this method is to systematically reduce the content into categories while maintaining its complexity [24]. In vivo codes were used to capture the content of the text which was then condensed into groups to increase traceability and confirmability of the results [25]. The final coding system was created abductively [26, 27] by means of two revising analyses and one final analysis cycle of all data: consisting of a database of 133 codes divided into 12 main categories. Some of these main categories include subcategories. The resulting categories were formed in parallel, i.e., inductively, guided by the codes, and deductively, beginning with the research question. Each code and category were then described and defined in a standardised format in order to maintain the intersubjective neutrality. If a text passage could not be unambiguously assigned to a code, the selected code was justified by a comment.

The trustworthiness [28] of the analytic outcome was ensured through discussions with two academics with expertise in qualitative research. Both were engaged in other subprojects of the overall research project and therefore familiar with the data set [16, 17].

## Results

Findings revealed three dimensions of spiritual care within which commonalities and differences occurred: function (evaluating the need / request to discuss spiritual issues), structure (evaluating when / how to discuss spiritual issues) and context (evaluating why / under which circumstances to discuss spiritual issues) (Table 3).

**Table 3:** Results – Overview

Aspects of Spiritual Care		HCP about HCP	HCP about CPP	CPP about HCP	CPP about CPP
**Function**	**Deontic Aspects (Need to discuss spiritual issues)**	Healing on a spiritual-psychological level is important. However, there are more important aspects in the structuring of the conversation.	CPP expect treatment primarily in a mechanistic-scientific manner.	HCP should be more open to non-medical concerns.	CPP want to be perceived as integral human beings.
	**Voluntaristic Aspects** **(Request to discuss spiritual aspects issues)**	HCP tend not to deal with spiritual issues or to express effort in this regard.	CPP would rather not talk to HCP about spiritual issues.	HCP should pay more attention to spiritual (and related) needs.	CPP want to have the opportunity to talk with HCP about spiritual issues.
**Structure**	**Temporal Aspects** **(When to discuss spiritual issues)**	Initiative for a discussion about spiritual issues must come from CPP - but generally still comes from HCP.	Religious-spiritual needs should be clarified as early as possible - possibly as soon as in the first contact. However, the exact timing must be determined individually.	Religious-spiritual needs should be clarified as early as possible. However, the exact timing must be determined individually.	Initiative for a conversation about spiritual issues must come from CPP or HCP - but generally comes from CPP.
	**Modal Aspects** **(How to discuss spiritual issues)**	The question of spiritual needs should be asked as openly as possible - but the transition to the conversation makes the HCP feel insecure, as does the conversation itself.An openness on the part of the HCP with regard to other attitudes is essential.	CPP want spiritual-religious help. This can, but need not necessarily, be conducted from a neutral point of view.	HCP are challenged in conversations about spiritual matters. In the conversation, the HCP should remain neutral and open regarding other attitudes of life.	CPP are grateful for religious-spiritual help, if desired.
**Context**	**Causal Aspects** **(Why to discuss spiritual issues)**	Enduring the situation together with the CPP is part of the therapy - spiritual issues can / could be directly integrated into the treatment of the CPP. HCP must understand CPP in order to contribute optimally to healing.	Spirituality is an important aspect of chronic pain: Spiritual interpretation of chronic pain can positively or negatively influence suffering and the healing process.	Enduring the situation together with the CPP is part of the therapy. HCP should indicate (spiritual) resources and enable a link between medicine and spirituality.HCP must understand CPP in order to contribute optimally to healing.	Spirituality is an important aspect of chronic pain: Chronic pain affects people in many different areas of life. Spiritual issues are an important, healing support.
	**Conditional Aspects** **(Under which circumstances to discuss spiritual issues)**	Active willingness to engage in spiritual dialogue must be demonstrated on a basis of trust.	For meaningful therapy, CPP must have realistic healing expectations and honestly express their needs.	HCP must actively demonstrate a willingness to engage in spiritual conversations on a basis of trust and be able to endure the situation along with the patient.	For meaningful conversations about spiritual issues, CPP must be reflective, open and honest.

### Function > Deontic aspects: evaluating the need to discuss spiritual issues

HCPs tend to assume that CPPs expect mechanistic and scientific solutions and want to be „repaired “as quickly as possible. They generally express the opinion that there are more pressing topics than spiritual issues when talking to CPPs. CPPs, in turn, tend to stress the importance of treating chronic pain not only on a physical but on a mental-psychological level as well. Some mention spiritual issues as a factor they have neglected for a long time, but which gains in importance in their current health situation. This divergence in weighting the importance of talking about spiritual issues was partly responsible for the general disappointment that CPPs reported from their contact with HCPs.

It should be noted that not all CPPs claimed that spiritual issues were important to them and something they wanted to have respected by HCPs or other third parties. Nonetheless, CPPs clearly and specifically expressed the desire for more openness from HCPs, by which they meant HCPs having the ability to admit treatment mistakes, communicate limits of their own knowledge and respond to the chronic pain patient’s request for alternative medical or spiritual explanations and healing approaches for their chronic pain. Perhaps most important was CPPs’ need to be accepted, perceived and taken seriously as an individual (spiritual) *entity* rather than being perceived merely as a patient with solely bodily suffering. CPPs often voiced this need as being central to their process of *healing*.

For CPPs, the concept of *healing* in this context meant achieving a state that is physically and psychologically bearable; a state that they can accept, and which ultimately allows them to perceive their lives as worth living – even if it may mean living with chronic pain symptoms (Table 4).

**Table 4:** Anchor Example – Evaluating the need to discuss spiritual issues

	Anchor example
**CPP**	Because of what... he is also only a human being, he has an education in things you can see, you can measure, and we don’t have that. And that is why the topic of spirituality and existence is EVEN more important! Because only if we understand ourselves and also the doctor understands that he cannot help us in the traditional sense. Because the rage you feel towards the doctors makes us sick, too. And we can’t solve that anyway. The doctors can’t learn anything more in these medical schools than they already do. And our problems may be understood in a hundred years - maybe not, but I think we also need some help to do that... that is a big part for me - the acceptance that nobody can help me makes me hurt the most - emotionally. Because I know I’m relying on myself, actually. And that makes fear and there it would be nice the fear, could be addressed, just at the beginning, when you arrive, not ah why are they crying, what is going on? Yes hello I’m in a hospital, I didn’t expect to end up in a hospital at thirty-two. Can you please have a little empathy? And ask me: What is important now so that I feel comfortable there? That I’m off to a good start. And don’t treat it like number 214. This would be important. (1.1_CPP | 00:41:15)
**HCP**	They come to us with expectations: either they are now in the right place, where they can now be provided with technical or medical … medically or in any other form of support. They do not come to us in the expectation: now with us... to discuss spiritual things with us... Because they’re being referred by the spec... well, they are referred to us by specialists for pain and pain management. And I believe the expectation which they have of us, that’s not on a spiritual level. (4.2_HCP | 00:12:59)

### Function > Voluntaristic aspects: evaluating the request to discuss spiritual issues

Both CPPs and HCPs found it easy to discuss spiritual issues within their own peer groups – the difficulty arose in the interaction between the two. CPPs repeatedly and clearly expressed a desire to talk to HCPs about spiritual issues. This ranged from showing subtle signs of being generally open to the topic to relating to their long-term illness narratives. Those CPPs regarded it as crucial that their spiritual needs be acknowledged by the HCP, stating that spiritual issues should be given more attention.

CPPs frequently described the desire for being perceived as an individual human entity and being cared for by HCPs; CPPs conveyed this as a form of emotional support independent of religion and many related this closely to spirituality. A majority of CPPs wished for spiritual issues to be addressed or at least be a potential topic to be explored during interactions with HCPs.

Those who preferred not to discuss spiritual issues with the HCP had different expectations of HCPs, wanted to take care of their spiritual needs themselves, or did not consider spirituality to be of importance (Table 5).

**Table 5:** Anchor Example – Evaluating the request to discuss spiritual aspects

	Anchor example
**CPP**	- Never. Never with me. Well, it’s never been discussed. I’ve been in treatment for years, but just - I’ve never mentioned the topics, that’s a bit - but it’s good to think about it today. (smiles) Maybe someone has experienced this. I only speak for myself. (1.1_CPP | 00:16:07) - Okay. Okay. You did shake your head as well. (1.1_CPP | 00:16:09) - No, I have not experienced it and I would be glad if I did not constantly have to seek help myself. If there were a person present where I could just walk up to them: Let’s talk. That - quite simple. (1.1_CPP | 00:16:22)
**HCP**	- No, if I have the choice between examining the patient and somehow go into depth with spiritual, then I will rather look at the place of pain. (4.2_HCP | 00:08:00) - Yes, but that they mention it spontaneously … I mean, I have known people since - I don’t know - since I am here, who always come back, where I would have NO idea what they are going to... how they got to the whole... what they think about this stuff, so even though I’ve had so much time with them. It’s just like - it comes... it’s not automatic. I think it’s... well, that’s my experience. But - (4.2_HCP | 00:08:29)

### Structure > Temporal aspects: evaluating when to discuss spiritual issues

CPPs and HCPs shared the view that spiritual needs should be addressed as early as possible in the treatment process. However, it was almost exclusively HCPs who stated, in a few instances, that this should be broached during the first contact. Both groups noted that trust should be established prior to talking about spiritual aspects. Much variance emerged regarding who should take the initiative to start a spiritual-religious conversation:

Both CPPs and HCPs stated that CPPs must take the first step by indicating an interest in talking about spiritual issues – partly because it has or can potentially be perceived as a taboo subject. HCPs felt it was not their place to broach the subject, while CPPs considered this an option. CPPs imparted that not all of them felt empowered to address the topic the way they would like to.

Striking discrepancies were found in the frequency of more general examples given in the interviews as to who - CPPs or HCPs - had or should have addressed spiritual issues: Both groups described approximately the same frequency with which the topic was addressed by CPPs. Statements that ascribed addressing spiritual issues to HCPs, however, occurred much more often amongst HCPs.

### Structure > Modal aspects: evaluating how to discuss spiritual issues

There was broad agreement between HCPs and CPPs that spiritual issues can be a sensitive subject not everyone feels comfortable talking about. HCPs related that it is usually they who direct a conversation towards spiritual issues, if at all. According to the HCPs, an indirect approach should be used by asking an open question, e.g. about resources. CPPs, on the other hand, did not tend to address the aspect of difficulties in broaching the subject.

This divergence between the two groups may be explained by the fact that HCPs felt uncomfortable with spiritual-religious discussions. They described the work with chronic pain patients as rather demanding. The CPPs sensed the HCPs’ uncertainty. They frequently described incidents where spiritual issues were addressed at the wrong time or in the wrong way and communication failed. However, the CPPs did not solely blame the HCPs. Instead, they advocated for mutual openness.

Opinions between the groups diverged on the question of whether and to what extent HCPs should take a neutral stance in discussions about spiritual issues. The predominant opinion amongst HCPs was that a strict neutrality on their part might be relinquished partially or completely in order to give advice, suggestions or tips (e.g. Table 6, HCP). CPPs were very grateful for these “suggestions” - however, it was more important to them than to the HCPs that HCPs not express their own affiliation or spiritual beliefs.

**Table 6:** Anchor Example – Evaluating when to discuss spiritual issues

	Anchor example
**CPP**	As such on the one hand - if now the patient knows that he can address that himself. And that might be written somewhere. Mm - (thinks) so yes I wouldn’t exactly address that at the first visit. If if... if, for example, you were to think about it now, the physiotherapist would treat the subject now, that he would get to know you... first of all this way and then you slowly get to know it a little bit. I could now imagine that the physiotherapist himself would notice a little bit: ah yes, now I could also slowly address the subject. Simply out of the feeling, where then in the course of time arises, with the patient together. (5.1.1_CPP | 00:33:26)
**HCP**	There are patients, I would certainly not do this the first time or maybe not at all. Because I realise I’m not the one who has to open this spiritual window with him. But when I realise that I can build up a certain connection to the patient, then I address that immediately. Yeah, because I think the sooner the better. (5.2_HCP | 00:31:21)

When touching upon and talking about spiritual issues, both parties stressed the necessity for the HCPs to be open to beliefs other than their own. To HCPs and CPPs, openness meant observation rather than interpretation, tolerance rather than defense, and appreciation rather than indifference.

Both groups shared the opinion that healthcare professionals have to be aware of their own spiritual-religious beliefs when talking about spiritual issues with patients.

Furthermore, some of the CPPs wanted more interprofessional collaboration from staff when gathering information about patients, be it spiritual or non-spiritual.

With few exceptions, neither group saw the need to include healthcare chaplains in the therapeutic team. Nor did they consider a specific questionnaire necessary to facilitate a conversation about spiritual issues (Table 7).

**Table 7:** Anchor Example – Evaluating how to discuss spiritual issues

	Anchor example
**CPP**	That’s why I am - yes, that’s why I am who I am or and that - yes, you experience a lot with the doctors simply then, a bit on the other side. So the subject has already been addressed with me. Just a little bit - maybe just then not exactly where I just wanted it to be. (2.1_CPP | 00:23:33)
**HCP**	- Maybe you should believe in what you believe in, whatever that is, if someone has chronic pain, maybe you should tell them that they need something … maybe something could be done about that, like a slight change so that maybe it would be better with the pain. (1.2_HCP | 01:11:22) - Yes, but it’s difficult to do that without being overbearing and missionary. (1.2_HCP | 01:11:29) - If you don’t believe in a punishing God, then maybe something could be said like he might not be so punishing. (1.2_HCP | 01:11:37) - Yeah, yeah, that’s what I’m saying. (1.2_HCP | 01:11:44)

### Context > Causal aspects: evaluating why to discuss spiritual issues

Chronic pain was sometimes interpreted by CPPs as a kind of “emergency brake of the body”, in a certain way allowing it to be explained, or even accepted and endured. It was much more common to describe chronic pain as excluding them from everyday social life and causing fear, anger and sadness. They felt vulnerable and often trapped in a vicious circle of constant pain and emotional upheaval.

In addition to the existential questions associated with financial hardship, job or housing loss, suicidal tendencies may arise with CPPs. For CPPs, the effects of pain were experienced not only psychologically and physically, but also on a spiritual level. For some, chronic pain led to questions about the meaning of life, or meaning in general, and destabilised or transformed their entire belief system.

HCPs perceived both the positive and negative effects of suffering, and acknowledged the significance given to the spiritual by CPPs when interpreting their situation.

Although both aspects of spirituality were considered, HCPs and CPPs mainly focused on its positive aspects. In their view, spiritual and religious beliefs were a resource for illness management. Spirituality creates community and support or can serve as an explanation for suffering during periods of feeling utter helplessness. Some of the statements indicated that CPPs experienced spiritual faith as healing.

HCPs and CPPs shared the conviction that such healing requires faith in the possibility of healing itself. Both groups were convinced that spirituality is an important factor in chronic pain and its therapy.

Many CPPs perceived the capability of HCPs to bear witness to their situation. They saw HCPs’ role as a supportive one and an inherent part of the therapeutic process. They wanted the HCP to draw upon their own resources and create a bridge between medical and spiritual support. Several HCPs confirmed the CPPs’ conviction that spiritual issues could be integrated directly into treatment.

The HCP’s role at the side of the chronic pain patient, bearing witness to their situation and conveying sincere empathy remains the most important aspect of spirituality in the treatment of chronic pain. This is a pivotal factor contributing to optimal healing - a point made by both groups, but emphasised by the CPPs (Table 8).

**Table 8:** Anchor Example – Evaluating why to discuss spiritual issues

	Anchor example
**CPP**	I would have had to have an operation this year and it just didn’t go well for me, but I was there for the talks, for the anaesthesiologist and so that’s unbelievable, he was so open, he immediately asked a few questions... he just knew where I stood, how I was and with the death of my daughter and then said: Yes he lost his partner 7 years ago so suddenly, by a sudden death. And then he just talked about himself and that’s so... that was INCREDIBLE and then he said: Look it’s important, I can just tell you that now from experience - that now we’ve just come out of the hole again and show ourselves in the village and again... so just like that! And that’s what... so this has been incredible how this has helped me. And he said what helped him and so yes all... very special and he also had time. And the time... the time... time problem in modern medicine. (3.1_CPP | 00:57:49)
**HCP**	Or also when someone just talks about the pain and he is now in a hopelessness like in there but I also know that there is also a resource for someone, now for the patient, the faith, then I also often ask then: Yes, what do they think God is saying to you at that moment, in this situation? What kind of thoughts or that ehm can … is one … mostly … so if it is really a resource it is mostly very encouraging for the patients and otherwise it shows a lot about the disease model they have. So when they say: yes, God wants me to suffer now - then that is very important information. (2.2_HCP | 01:03:53)

### Context > Conditional aspects: evaluating under which circumstances to discuss spiritual issues

One factor that often seems to prevent a conversation about spiritual issues is time: HCPs and CPPs agreed that religious-spiritual conversations need time, which is often lacking.

Some HCPs noted that non-religious spirituality was difficult for them to address in conversations with CPPs. They not only wished for more time, but also for better guidance - two points which seem to be exacerbated by their general discomfort regarding the topic.

CPPs frequently mentioned a preference for certain professional groups of HCPs when discussing spiritual concerns. Specifically mentioned were psychiatrists, anaesthetists, nurses and physiotherapists. This underlines the importance of interprofessional collaboration in integrating spiritual issues into the treatment process.

Before a discussion on spiritual issues can start, a basis of trust must be established in which the CPPs feels safe to open up to the HPC, as stated above. This is necessary because personal spirituality can potentially be considered a taboo subject. This was stated clearly by both groups.

Such a basis of trust arises from the CPP’s perception of being taken seriously by HCPs who are interested in them as a self-determining individual, a person as a whole. HCPs can create a feeling of security that enables trust to be established.

To build on this basis of trust, HCPs must be able to actively show a willingness to discuss spiritual issues - both groups again agreed on this point. Showing active willingness involves signalling verbally as well as non-verbally that spiritual issues can be talked about. It means showing unbiased openness and curiosity as well as responding even to subtle signals of the CPP. The CPP, were also well aware that a meaningful conversation about spiritual issues requires self-reflection, openness and honesty on their part (Table 9).

**Table 9:** Anchor Example – Evaluating under which circumstances to discuss spiritual issues

	Anchor example
**CPP**	You notice it in people - the hectic pace. So - if you can’t even look each other in the eye today. When there’s not even time left to look at someone. Or say hello. Give me a smile. Time’s gone, that’s sad but true. How then WILL one - meet another person on the spiritual level? Well, I-I think that’s impossible. There should really be another sensitising FROM patients themselves as well. So from all people. That you can do that. That one - so yes - (4.1.1_CPP | 00:55:24)
**HCP**	- I think they also have to bring along an acceptance that maybe the pain afterwards is just as strong as before. So that they come here and say: I want my pain to go away now that the thirtieth clinic is here. And they have had them for maybe 10 years, then I probably won’t take them in. So in the clarification conversation. It’s... you have to be willing to talk about how to deal with the pain. And if you just want to make it go away, it’s just frustrating for everyone. Because faith has something to do with managing pain. If you’re not willing to talk about it, it’ll be a standstill. (chuckles) (2.2_HCP | 01:08:02) - Only the demand, effectively, so if right there... so only the demands on us. I want that... if I’m... if I’m coming up with... with ideas - what do they expect after they’re released, how should it go. Pain scale from for example... from seven eight, to two. Or is... well, that’s almost unrealistic. Um, these things... just, their willingness to redefine trust in all areas of life. (2.2_HCP | 01:08:58)

## Discussion

Our results show that, in general, CPPs’ and HCPs’ views on spiritual concerns and needs concur. However, significant differences were identified in their expectations and ideas about when and how to raise spiritual concerns and needs within the therapeutic process. This might partially explain why spiritual issues are not adequately addressed in current therapeutic approaches to chronic pain [9].

Another factor that contributes to the neglect of spiritual issues in the current treatment of chronic pain might be the manifold understandings of *spirituality* in and among both groups: Considering spirituality as a dynamic dimension [29] or a *travelling concept* [30], allows for a more inclusive definition, which is a necessity in the health care context [21, 22]. Nevertheless, the term *spirituality* in itself might not always be accepted by non-religious people to be inherent in human life itself.

Divergent perceptions of *spirituality* are mirrored in the interviews, in which some participants interpreted certain activities or experiences such as making music or experiencing nature as distinct from spirituality, whereas other participants viewed those as manifestations of spirituality. Furthermore, many issues individually described as spiritual might not seem so to others, e.g., pragmatism, materialism, the pain itself, or the mere feeling of being understood and taken care of, as experienced by the chronic pain patient during interactions with the HCP.

Overall, *spirituality* was used by the interview participants as an umbrella term for activities and experiences that support the process of sense-making, independent of religion. In this way, spirituality can support CPPs in their endeavor to endure a seemingly senseless situation and to ease personal suffering.

These findings are in line with the literature, where spiritual issues were found to be a possible resource in [11, 12, 14], or to have an impact on the treatment of chronic pain [15]. The neglect of spiritual issues might be one factor of the dissatisfaction with treatment so frequently expressed by CPPs [1–3].

Based on our data, six key insights to improve the therapeutic situation for CPPs and HCPs can be suggested (Table 10).

**Table 10:** Discussion – Key insights

Aspects		Key insights
**Function**	**Deontic**	CPPs want to be perceived in the entirety of their (spiritual) integrity as human beings in HCPs treatment of them
	**Voluntaristic**	A large number of CPPs considers spiritual issues in therapy to be insufficiently recognised.
**Structure**	**Temporal**	The two groups hold diverging opinions as to whether it is the CPP or HCP who should take, must take or actually take the initiative for a discussion on spiritual issues.
	**Modal**	HCPs do not feel well prepared to initiate and to discuss spiritual issues.
**Context**	**Causal**	In therapy, HCPs must be able to bear witness to the CPP’s situation at their side.
	**Conditional**	Mutual trust is necessary for a shared approach in general and for a conversation about spiritual issues in particular.

Transferring these recommendations to a larger context, three ranges of action in integrating CPPs’ spiritual needs and concerns in current health care can be suggested:

- *Making room for spiritual needs*: To date, the aspect of spirituality has received little to no attention in chronic pain therapy [9], leading to current treatment being described as generally unsatisfactory by most CPPs [1–3]. Ideally, the CPP’s interest in including spiritual issues should be ascertained as soon as possible, so that it can serve as a resource (as mentioned in [11, 12, 14, 15]) within therapy whenever appropriate. However, as spirituality is potentially a taboo subject requiring a certain level of trust for an interaction – regardless of the HCP’s professional affiliation – the enquiry must be made in a non-threatening manner. One possibility would be to evaluate the matter within the framework of a status enquiry and anamnesis at the time of admission, in writing or verbally. Corresponding questions would have to be carefully chosen and examined for three distinct reasons: there is a) broad conceptuality in the manifold definition of spirituality, b) a lack of a generally accepted definition in the general population, and c) an issue for some individuals who object to the term spirituality itself, more than what it embodies or can embody.
- *Initiate the talk*: The skill of initiating and guiding conversations is also essential in the effort to support the patient on a spiritual level. HCPs voice doubts when it comes to conversations on spiritual issues. Furthermore, both CPPs and HCPs share the opinion that discussions on spiritual issues require training, which they lack – a finding that underscores the relevance of pre-existing studies [4, 10]. The first step would be to make HCPs more aware of the relevance of spiritual issues as an aspect of care. In the longer term, a more extensive training for HCPs to address spiritual issues would be useful [31]. HCPs should prepare themselves through training and continuing education to gain confidence in assessing and addressing these concerns and needs in an adequate setting, time and space.
- *Meet halfway*: Chronic pain is a burden. Its treatment requires both health professional and patient to be approachable, and, to some extent, step out of their traditionally assigned roles in the healthcare setting in a joint effort to work closely with chaplains for specialized spiritual care. In order to meet halfway and to improve the current situation regarding the treatment of chronic pain, CPPs need to be guided in expressing their (spiritual) needs in such a way that the HCP can understand them. Improvement can be brought about by assisting chronic pain patients to learn to accept their situation to the extent that they are able. This would include their approaching the HCP with the hope of relief – more than with the hope of absolute healing. It would also be positive if the patient could be helped to the awareness that their situation often presents a difficult-to-manage therapeutic situation HCPs as well. HCPs should become more aware of the relevance that spiritual concerns and needs can have for CPPs in their pain management. Their role in bearing witness to the chronic pain patient’s suffering can support them in the process of *healing*.

### Strengths and limitations

This paper contributes to the growing relevance of the spiritual dimension in health care. The multi-centre approach regarding data collection taken in this study relativised potential differences between facilities. It was strengthened by the interviews with HCPs and CPPs being conducted separately, which allowed for triangulation of the data sources. Having the interviews moderated by a nurse scientist and a theologian with an expertise in biomedical ethics enabled a balanced and comprehensive exploration of the topic in the course of discussion and reduced the risk of interview bias. The coding system for analysis was generated in several inductive and deductive processes. Qualitative content analysis with documentation principles enabled a critical examination of results to be rooted in the original data. Frequent discussions with two qualitative researchers supported the trustworthiness of the results.

A limitation of the study is that the audio transcripts did not reflect non- and paraverbal aspects of care which would have offered additional information on participants’ views. Furthermore, the sampling procedure might have introduced a selection bias resulting in an overrepresentation of participants with overly positive attitudes towards spirituality. Also, the term *existential* was added to the definition of spirituality in order to include patients who would not consider themselves to be spiritual. This led to some CPPs to interpret this term financially, because of pressing monetary concerns they had themselves experienced.

## Conclusions

In this study, we developed six key insights (Table 10) leading to three different and distinct potential ranges of action: Spiritual issues need a) a carefully chosen framework in which they can be discussed, b) training or continuing education in order for HCPs to appropriately initiate the talk about spiritual issues and c) willingness by both parties, HCPs and CPPs, to meet halfway in order for these issues to be thoroughly discussed.

The key insights may explain in part why the spiritual dimension is remains neglected in the treatment of chronic pain.

A major hindrance to the integration of the spiritual dimension is the time factor – and therefore, indirectly, money. The extent to which these ranges of action also make economic sense would have to be examined in a cost-benefit analysis focusing on whether the positive effects of this training for HCPs and the resultant intervention outweigh its costs in terms of efficiency. While the economic implications have yet to be clarified, it can be said that patients need partners in their therapeutic process, i.e. health care professionals who are open to what is most significant in a patient’s life, be it of a physical, psychological, social or spiritual nature.

## Data Availability

The datasets generated and analysed during the current study are not publicly available due to restrictions related to patient confidentiality and limitations of patient consent forms but are available from SPK upon reasonable request.

## Abbreviations

CPP: Chronic Pain Patient
HCP: Health Care Professional
